# Vascular Epiphyte Diversity Differs with Host Crown Zone and Diameter, but Not Orientation in a Tropical Cloud Forest

**DOI:** 10.1371/journal.pone.0158548

**Published:** 2016-07-08

**Authors:** Xixi Wang, Wenxing Long, Brandon S. Schamp, Xiaobo Yang, Yong Kang, Zhixu Xie, Menghui Xiong

**Affiliations:** 1 Key Laboratory of Protection and Development Utilization of Tropical Crop Germplasm Resource, Ministry of Education; College of Horticulture and Landscape Agriculture, Hainan University, Haikou, People’s Republic of China; 2 Department of Biology, Algoma University, Sault Ste. Marie, Ontario, P6A 2G4 Canada; Chinese Academy of Forestry, CHINA

## Abstract

Vascular epiphytes are important components of biological diversity in tropical forests. We measured the species richness and abundance of vascular epiphytes along four vertical crown zones and five horizontal orientations on 376 trees, as well as the diameter at breast height (DBH) of host trees in tropical cloud forests in Bawangling, Hainan, China. The relationship between vascular epiphyte species richness and host tree DBH was assessed using a generalized linear model. There were 1,453 vascular individual epiphytes attributed to 9 families, 24 genera and 35 species, with orchids and pteridophytes dominating. Both the species richness and abundance of epiphytes significantly differed among the four crown zones for all collections and each host tree, suggesting that vertical microhabitats contribute to the distribution of epiphytes on host trees. Neither epiphyte abundance nor species richness differed among the eastern, southern, western, and northern orientations for all host trees; however, both richness and abundance were significantly higher for epiphytes that encircled host tree trunks. This suggests that morphological and physiological characteristics of the tree, but not microclimates probably contribute to the distribution of epiphytes on host trees. Epiphyte species richness was positively correlated with tree DBH across the six host tree species studied, with increases in DBH among smaller trees resulting in larger increases in richness, while increases in DBH among larger host trees resulting in more modest increases in ephiphyte richness. Our findings contribute support for a positive relationship between epiphyte species richness and host tree DBH and provide important guidance for future surveys of epiphyte community development.

## Introduction

Epiphytes are essential components of biological diversity that germinate and grow upon host plants (typically woody perennials), obtaining mineral nutrients and water from water vapour (e.g., fog) during at least part of their life history [1,2]. There are approximately 20,000 species of vascular epiphytes accounting for 10% of all vascular plants [1]. In tropical vegetation, however, vascular epiphytes make up around 25% of vascular plant species [3] and play important roles in maintaining forest ecosystem functions (e.g., nitrogen fixation, water and nutrient cycle) [4,5].

As trees grow, their morphological and physiological characteristics, including tree architecture [6], bark roughness [7], canopy soil chemistry [8], and branch inclination [9] often influence epiphyte community development [2]. But many studies show that epiphytes are associated with crown height [10,11] and orientation of host trees in relation to associated variability in microclimatic conditions [12]. On host trees, vertical environmental heterogeneity in humidity, temperature, the accumulation of organic matter, and solar irradiance, have been found to be responsible for epiphyte distribution in relation to tree crown height [13]. For example, Adriano & Mário [14] found that epiphyte richness and abundance were greatest on host trunks in the lower or upper crown zones, due to variation in light and moisture which form a microclimatic gradient along the length of the host tree. Additionally, differences in microclimatic gradients related to orientation caused by sunlight intensity and wind strength, may have an effect on the distribution of vascular epiphytes [12].

Trees can be considered discrete ecological units with fixed borders surrounded by different environments [15], much like islands. A logical extension of species-area relationships leads to a basic prediction that larger trees will support more epiphytes than smaller ones. Studies comparing epiphyte species richness across different environments or communities have generally addressed this by sampling trees of similar size or including tree size as a co-variate [16,17]. However, size, age, and environmental diversity of host trees change simultaneously over time, complicating the interpretation of their individual effects on epiphytic diversity [18]. Tree diameter, which is correlated with other tree characteristics such as bark structure, habitat complexity and tree architecture [19–21], is often taken as an independent variable, and has been used as a surrogate measure of both the tree size and age [22,23]. Relationships between epiphyte diversity and host tree diameter, however, remain very much a matter of debate, with two contrasting expectations existing in the literature. One prediction is that there should be a positive relationship between tree diameter and epiphyte species richness during a young tree's growth [24], while a neutral relationship would be observed for adult trees [22]. For young trees, the theory suggests that expanding diameter provides large areas with diverse microenvironments for the colonization and growth of epiphytes; while the rate at which such microenvironments accrue with increases in tree size diminishes in adult host trees, leading to a slower accumulation of epiphytes per unit size gained [25]. An alternate prediction is that there should be no relationship between epiphyte species richness and host tree diameter when trees are below the average diameter, as these younger host trees lack sufficient architectural and physiological characteristics suitable for epiphyte establishment [23]. The second part of this alternate prediction is that for trees above the average diameter, there will be a positive relationship between host tree diameter and epiphyte species richness because these larger trees provide more favourable microclimates for epiphyte colonization and growth [23]. Currently, results do not clearly distinguish between these two predictions; consequently, more research is needed to determine which set of predictions is more supported by data from natural communities, particularly in mixed tree-size forests.

Tropical cloud forests include all higher-elevation forests growing in the humid tropics of America, Africa and Asia that are frequently covered by cloud or mist [26,27]. Environmental conditions are characterized by low air temperatures, strong winds, frequent fog, and relatively high levels of ultraviolet radiation compared to conditions in tropical forests at lower altitudes [28]. Trees in tropical cloud forests are generally more deformed, primarily as a result of abiotic stresses in these habitats[29]. Tropical cloud forests possess a high diversity of epiphytes, including bryophytes, lichens, ferns, and seed plants that all contribute to the unique ecology [27,30]. Plant species in tropical cloud forests often experience stress related to low air temperature, wind exposure, and low soil phosphorus [29]. Negative and positive biological interactions have both been identified as influencing species coexistence in these ecosystems in accordance with the stress-gradient hypothesis [31–33].

In this study, we measured species richness and abundance of vascular epiphytes (epiphytes hereafter), as well as the diameter of 376 host trees spanning 25 20 × 20 m plots in tropical cloud forests in Bawangling in Hainan Island, Southern China. We used tree diameter as a surrogate for tree size and age to assess its relationship with epiphyte species richness and abundance. First, to understand what makes particular microhabitats favourable for epiphytes, we tested whether epiphytes were more frequently found in particular crown zones, or facing in particular directions on trees (orientation). Second, we tested whether the relationship between epiphyte species richness and host tree diameter varied with tree size in an effort to determine whether data from tropical cloud forests supports either of the two sets of predictions for this relationship from the literature.

## Materials and Methods

### Study site

This study was conducted in the tropical cloud forest (TCF) on Shifeng Mt., which is found in the Bawangling Nature Reserve(BNR), Hainan Island, Southern China. The survey in BNR was permitted be the Forest Department of Hainan Province. BNR is ca. 500 km^2^ in area, with altitudes ranging from ca. 100 m to 1654 m. The mean annual temperature is 23.6°C and the annual precipitation is 1677.1 mm at ca. 100 m altitudes, with a distinct wet season from May to October and a dry season from November to April [28]. The vegetation types are tropical lowland rain forest, tropical montane rain forest, and tropical cloud forest from the low to high elevation. This tropical cloud forest is composed of primary old growth forest (no history of human disturbance) spanning 0.40 km^2^, mainly distributed as mountaintop islands starting above altitudes of 1250 m. The mean daily air temperature in the rainy season (May-Oct.) ranges from 16.25°C to 20.57°C, and the mean daily relative humidity in the rainy season ranges from 87.88% to 100% [29]. The study forest is located on an eastern slope with the inclination ranging from 36° to 45°. It has an average tree height of 4.79 ± 2.80 m, a density of 9633 stems ha^-1^, a basal area of 54 m^2^ ha^-1^ and total of 139 tree species (trees ≥ 1 cm DBH). Dominant tree species include *Distylium racemosum* Sieb. & Zucc., *Symplocos poilanei* Guill., *Syzygium buxifolium* Hook. et Arn., *Cinnamomum tsoi* Allen, *Engelhardtia roxburghiana* Wall. and *Rhododendron moulmainense* Hook. f.

### Data collection

Twenty-five 20 × 20 m plots were randomly located within the central areas of the TCF forests (19°05'04.8" N, 109°12'43.5" E). In this study, we divided the 25 20 × 20 m plots into 400 25 m^2^ subplots. After measuring the DBH and crown height for all individual trees (excluding those clearly suckering from other trees) with diameter at breast height (DBH) ≥ 1 cm, we haphazard chose a tree in each 25 m^2^ subplot to survey for epiphytes, and there were 376 host trees spanning 48 species surveyed and we measured epiphyte species richness and abundance for each host tree.

The relatively small stature of host trees (i.e., 4.79 ± 2.80 m) in the study forest helped us to accurately measure species richness, abundance and orientation of all epiphytes on focal host trees. Host trees with a diameter at breast height (DBH) greater than 20 cm that had crowns that were not easily visible from the ground were climbed using single-rope climbing techniques (where deemed safe). Host trees with DBH smaller than 20 cm and height higher than 3 m were surveyed with binoculars and a thief rod [34], while small trees (height < 3 m) were surveyed from the ground. Samples of unknown epiphyte species were collected and brought back to lab for identification according using the Flora Republicae Popularis Sinicae [35]. In this study, clusters of epiphytic plants were identified according to the approach by Sanford [36]: if the intermingling individual stems belonging to conspecific plants were spatially separated and distinguishable from each other, these were classified as different individuals, while a collection of individuals consisting of more than one species, each was classified as different species.

**Fig 1:**
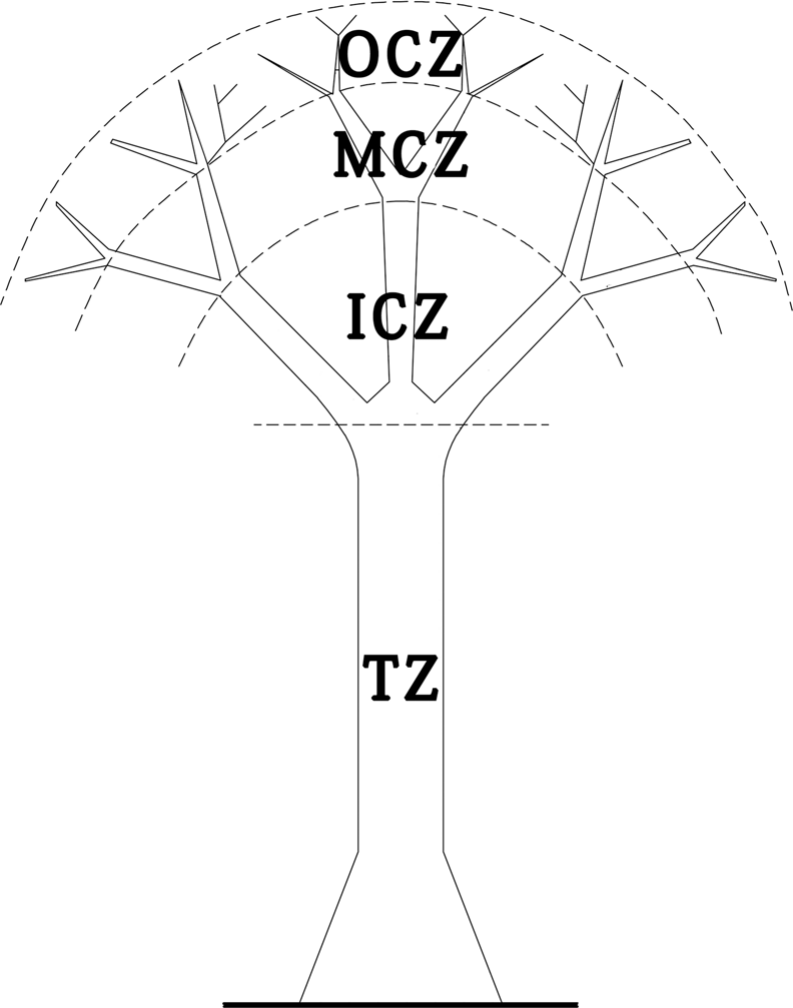
Diagram of the vertical zones of host trees where epiphyte is distributed. TZ, ICZ, MCZ and OCZ indicated trunk zones, inner crown zones, middle crown zones and outer crown zones of host trees, respectively. This disgram is redrawn from Johansson (1974).

Each investigated host tree was divided into four zones (Fig 1) [37]: trunk zone (TZ), inner crown zone (ICZ), middle crown zone (MCZ), and outer crown zone (OCZ). The TZ refers to the host trunk areas below the first branch; the ICZ covers the area from the first branch to the second branch; the MCZ covers the area from the second branch to the third branch; and the OCZ refers to the remaining areas above the third branch. The epiphytic orientations of epiphytes in each zone were classified into four directions: east, south, west and north, which indicates that epiphytes are found at only one of the four directions. Frequently, epiphytes encircled the trunk of the tree–we assigned such epiphytes to an “all directions” oriention. Epiphyte abundance and richness for the “all direction” orientation therefore refers to only those epiphytes encircling the trunk.

### Data analysis

Six host tree species with relatively high epiphyte richness and abundance (covering more than 50% of total epiphyte richness and abundance) were chosen and used to assess variation in species richness/abundance of vascular epiphytes among the four host crown zones and among the five orientation, respectively. First, considering host tree height and host tree identities likely affected species richness/abundance across all six host tree species, we assessed the effects of host tree height and host tree identities on species richness/abundance using a linear mixed-effects model in the R environment. For this test, host tree height is a fixed effect, while host tree identity was a random effect. Models were fit using maximum likelihood, and *Student’s* t-tests were used to assess the significance of the fixed effect in the model. We calculated the proportion of variance in epiphyte species richness and abundance explained by host tree identities. We found that host tree height did not significantly affect epiphyte species richness/abundance (S2 and S5 Tables), and host tree identities explained to less than 8% of the variance in epiphyte species richness/abundance (S2 and S5 Tables). Second, considering host tree height likely affected species richness/abundance for each the six host tree species, we assessed both the effects of host tree height and host crown zones on species richness/abundance using two-way ANOVAs. We found that host tree height did not significantly affect epiphyte species richness/abundance, while host crown zones did (S3 and S6 Tables). Finally, we examined the differences in species richness/abundance of epiphytes in different host crown zones and also among orientations on the hosts, respectively, using one-way ANOVAs, followed by multiple comparisons (Tukey-Kramer HSD tests). Epiphyte abundance data were rank transformed before all analyses.

We examined relationships between epiphyte species richness and tree diameter for each the six host species and across the six host species. Host tree size was approximated using DBH [22], which also caputres elements of tree ontogeny [23]. Patterns of epiphyte species richness often change with host tree ontogenic stages [22,23]; however, it is difficult to clearly distinguish the DBH boundary between these stages or the mix-sized forest tree species; for this reason, we took all individuals of each tree species together, and explored the relationships of vascular epiphyte species richness and host DBH using a regression with breakpoints (generalized linear model), with the segmented and nlme packages in R environments. Tree DBH was taken as an independent variable. The response variable (i.e., species richness), was assumed to follow a Poisson distribution, as is common with count data. All statistical analyses were performed with R 3.2.0 (R development core team) [38].

## Results

### Diversity of vascular epiphytes

There were a total of 1453 individual epiphytes, belonging to 35 species, 24 genera and 9 families (S1 Table). Epiphytic seed plants were most abundant, with 1139 individuals attributed to 23 species, 14 genera and 2 families. Epiphytic ferns were represented by 314 individuals spanning 12 species and epiphytic dicotyledons had the lowest representations with only 5 individuals of a single species.

The most speciose plant family represented by ephiphytes was the Orchidaceae, contributing 1134 individuals belonging to 22 species, with the genera *Dendrobium* and *Eria* having the highest number of species (s = 4). *Coelogyne fimbriata* had the most individuals present (n = 305), followed by *Bulbophyllum retusiusculum* (n = 204) and *Pyrrosia eberhardtii* (n = 149). Among epiphytic ferns, the Polypodiaceae was the dominant family, with 227 individuals, belonging to 5 species and 4 genera.

### Diversity of host trees

The surveyed 376 host trees were attributed to 48 species, 34 genera and 23 families, with their DBH ranging from 1.40 cm to 54.00 cm. *Hamamelidaceae* was the best represented family, with 110 individuals (29.30% of total). There were 200 host trees with less than five individual epiphytes and six host trees had more than 10 individual epiphytes (n = 11.30 ± 0.82). Tree species with relatively high epiphyte richness and abundance were *Distylium racemosum* (s = 26, n = 453), *Ternstroemia gymnanthera* (s = 19, n = 75), *Engelhardia roxburghiana* (s = 18, n = 84), *Cyclobalanopsis disciformis* (s = 14, n = 56), *Syzygium buxifolium* (s = 12, n = 86) and *Illicium ternstroemioides* (s = 10, n = 53).

### Distribution of vascular epiphytes among host crown zones

**Fig 2:**
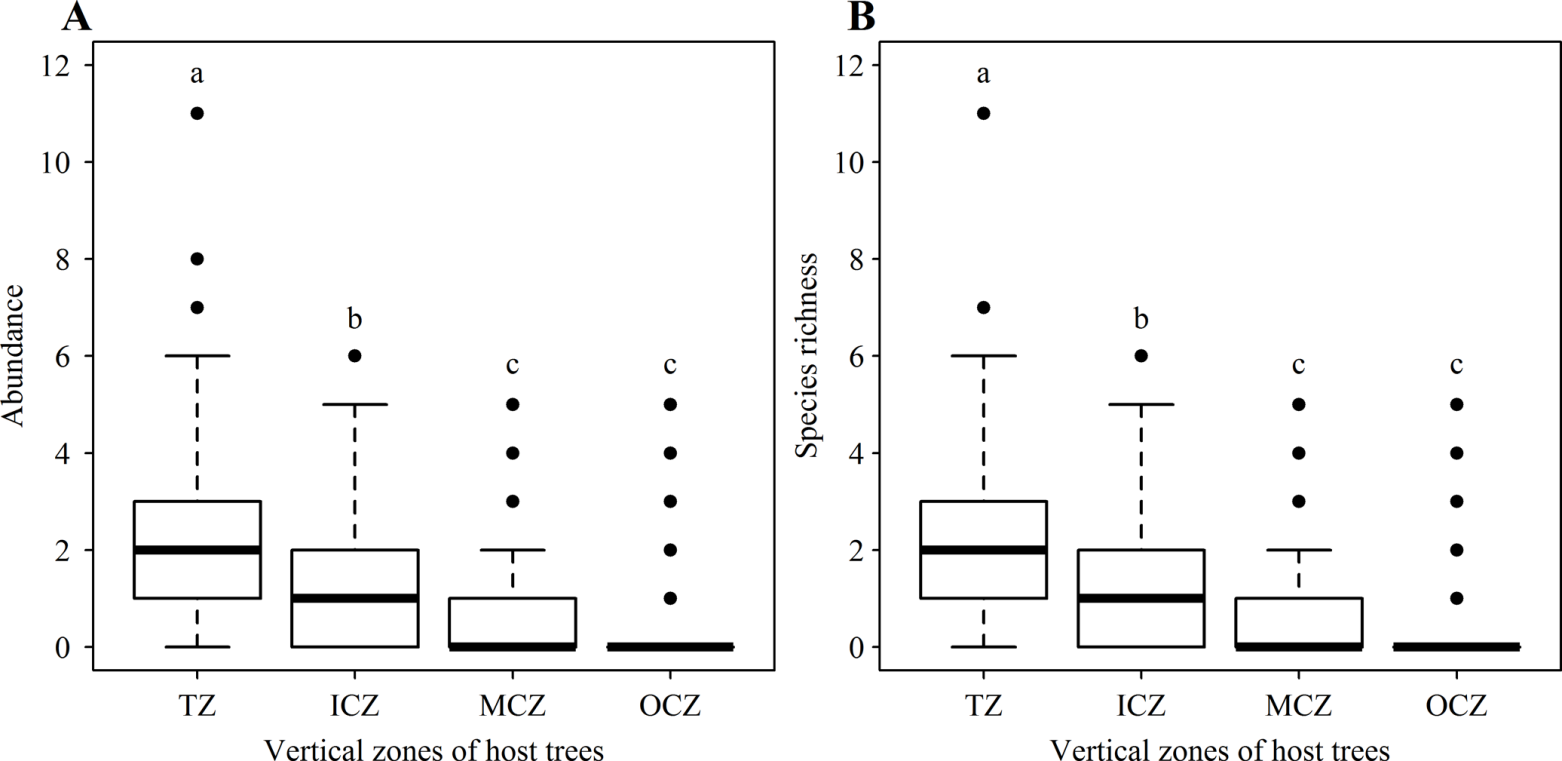
**Abundance (A) and species richness (B) of vascular epiphyte at the different crown zones upon host trees.** Different letters above boxes indicated significant difference for alpha = 0.05. TZ, ICZ, MCZ and OCZ indicated trunk zones, inner crown zones, middle crown zones and outer crown zones of host trees, respectively.

When host tree height was taken as a fixed effect, and tree identity was taken as a random effect, no obvious effects of host tree height on epiphyte abundance or species richness were found when all six host tree species were examined using a linear mixed-effects model (S2 Table). Host tree identity contributed to less than 8% of variance for epiphyte abundance or richness (S2 Table). Both epiphyte abundance and richness significantly decreased from the trunk zones (TZ), inner crown zones (ICZ) and middle crown zones (MCZ) to the outer crown zones (OCZ) of all host trees (epiphyte abundance, *F*_(3, 740)_ = 133.90, *P* < 0.001, Fig 2A; epiphyte species richness, *F*_(3, 740)_ = 126.30, *P* < 0.001, Fig 2B). Additionally, neither epiphyte abundance nor species richness were significantly affected by host tree height (S3 Table); however, both significantly differed among the four zones for each of the six tree species (S3 and S4 Tables).

### Distribution of vascular epiphytes among epiphytic orientations

**Fig 3:**
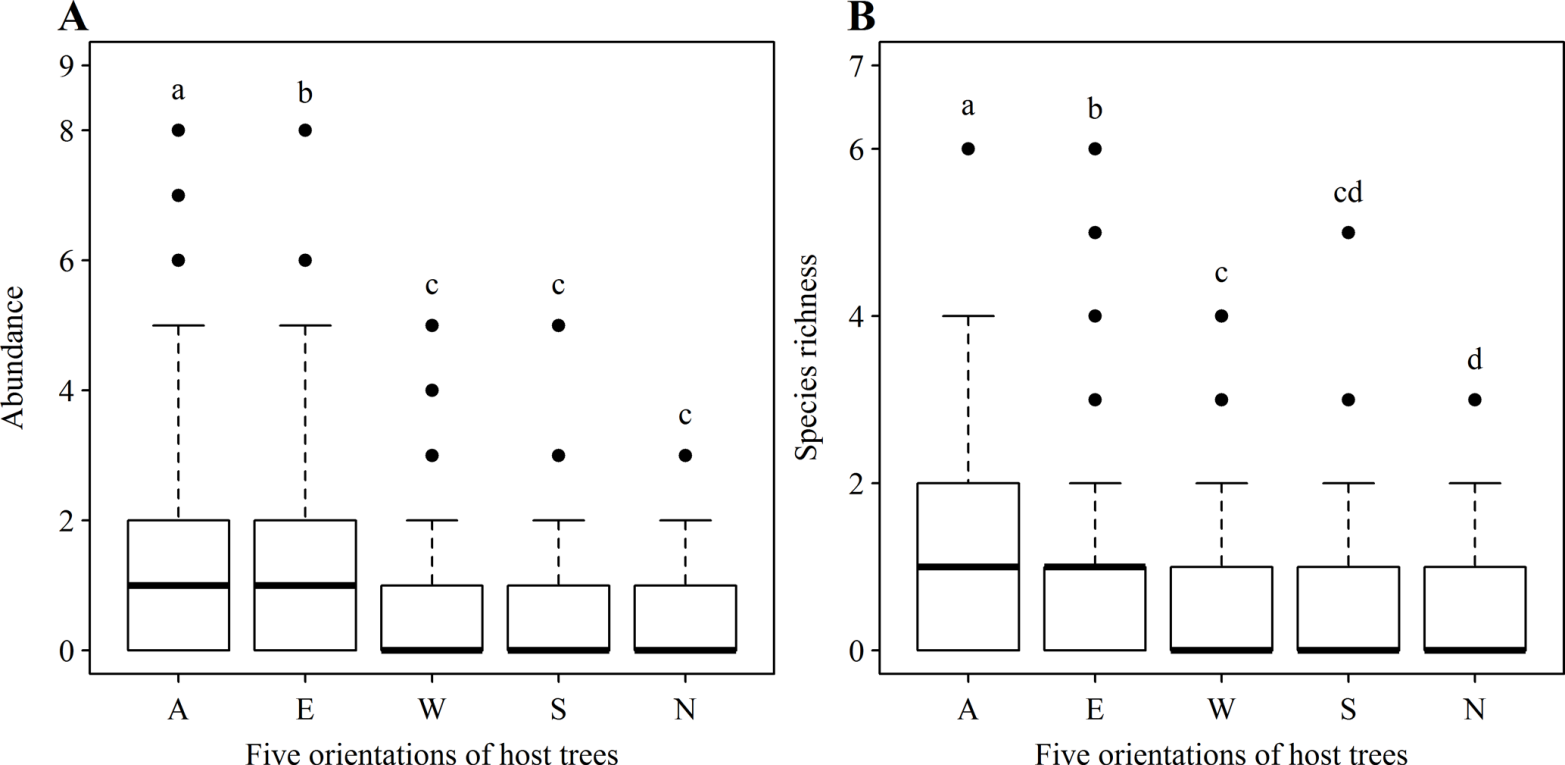
**Abundance (A) and species richness (B) of vascular epiphytes at the five orientations upon host trees.** Different letters above boxes indicated significant difference for alpha = 0.05. A, E, S, W and N indicated the five orientations of all directions, east, south, west and north where epiphytes grew upon host trees, respectively.

When host tree height was taken as a fixed effect, and tree identity was taken as a random effect, no obvious effects of host tree height on epiphyte abundance or species richness were found when all six host tree species were examined using a linear mixed-effects model (S5 Table). Host tree identity contributed to less than 9% of variance for epiphyte abundance or richness (S5 Table). Epiphyte abundance significantly differed among epiphytes which encircled the trunk (all direction orientation) and those that were oriented to the north (*F*_(4, 925)_ = 35.33, *P* < 0.001, Fig 3A). However, epiphyte species richness did not significantly differ across the five orientations (*F*_(4, 925)_ = 32.71, *P* < 0.001, Fig 3B). Moreover, when each tree species was considered separately, there were no significant effects of host tree height on epiphyte abundance and species richness (S6 Table). However, epiphyte abundance and species richness significantly differed among the five orientations for five of the six tree species (*Ternstroemia gymnanthera was the exception;* S7 Table).

### Relationships between vascular epiphyte species richness and host tree diameter

**Fig 4:**
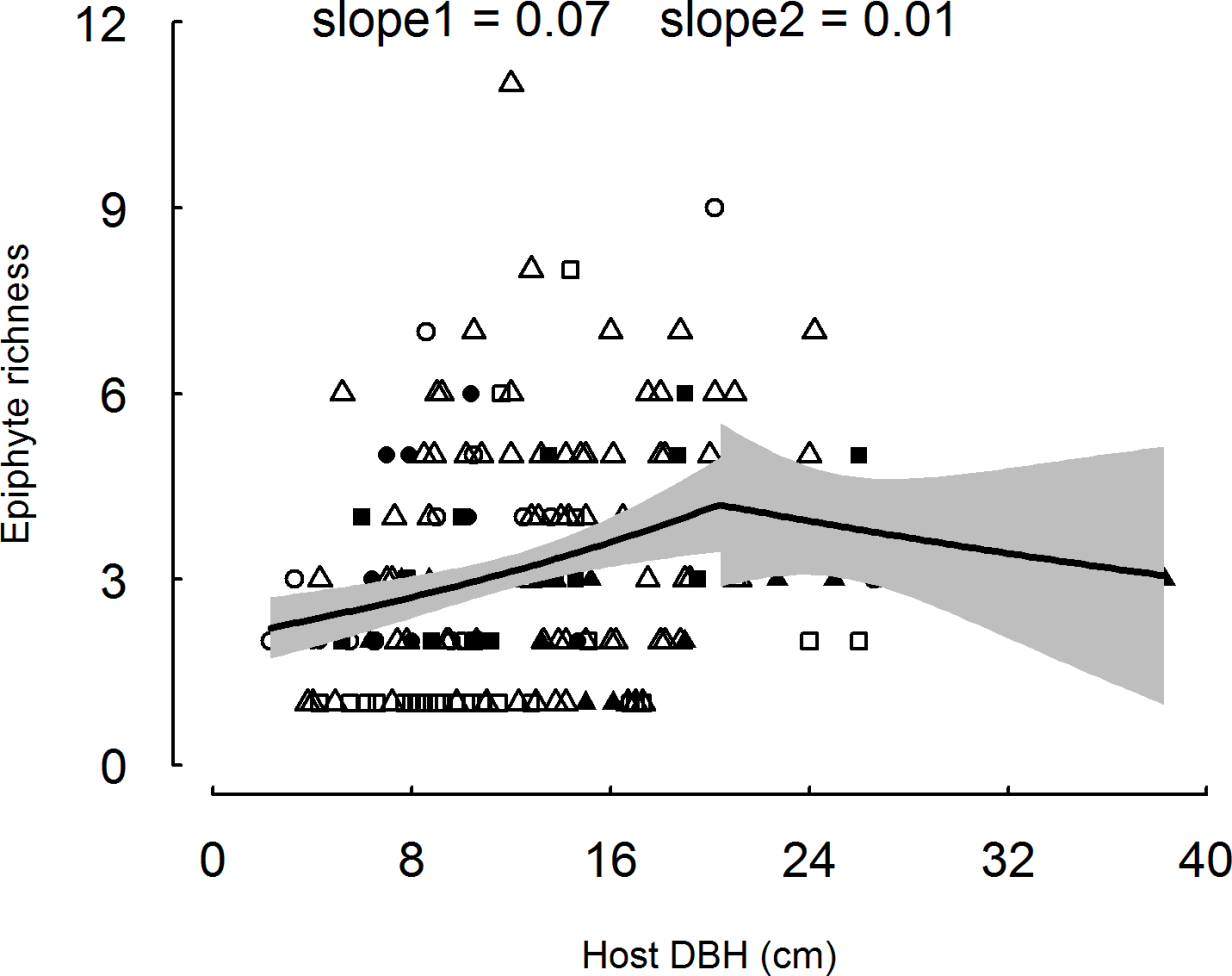
Relationship between the vascular epiphyte species richness and host tree diameter (DBH) across all six study species (*Engelhardtia roxburghiana*, *Ternstroemia gymnanthera*, *Syzygium buxifolium*, *Illicium ternstroemioides*, *Distylium racemosum* and *Cyclobalanopsis disciformis*), using a regression of breakpoints based on generalized linear model. Different symbols showed different host species. The lines represented locally weighted means and the shaded area represented 95% confidence interval.

When each of the six host tree species were analyzed independently, epiphyte species richness was not significantly related to host tree DBH, using a generalized linear model (S8 Table). However, when all species were grouped together, epiphyte richness increased significantly with increasing DBH (regression with breakpoints; P = 0.02). The slope of this increase was 0.07 when the host DBH was less than 11.98 cm and 0.01 when the host DBH was more than 11.98 cm (Fig 4).

## Discussion

### Diversity of vascular epiphytes

We found 1453 individuals and 35 species of epiphytes growing upon 376 individuals belonging to 48 species of host trees in 25 20 × 20 m plots of tropical cloud forests (S1 Table). Orchids and pteridophytes account for 99.66% of all epiphytes found in this habitat. Hainan Island is located at the northern margin of tropical Asia, and epiphytes in this region are characterized as both tropical and subtropical. Orchids are often the primary epiphytes in tropical rain forests [7,10,14]; while pteridophytes are a major group in subtropical forests [39]. The species composition by family observed in this study followed a worldwide trend of many species concentrated within Orchidaceae. For example, Boelter et al. [7], Adriano & Mário [14] and Zhao et al. [10] identified Orchidaceae as the dominant family in Brazilian Amazon tropical moist forest, in *restinga* forest in the north of Brazil and in a tropical montane forest in Xishuangbanna of Southwestern China, respectively. Epiphyte diversity in tropical cloud forests (35 species upon 376 hosts) is lower than those in Brazilian Amazon tropical moist forest (122 species upon 300 hosts) [7], Costa Rica tropical wet forest (97 species upon 61 hosts) [13] and Xishuangbanna tropical montane forest (103 species upon 77 hosts) [10], but greater than Brazilian Maracanã forest (11 species upon 254 hosts) [14], and Panama rainforest (77 species upon 173 hosts) [40].

### Distribution of vascular epiphytes among host crown zones and orientations

In our study, epiphyte species richness and abundance generally decreased from the TZ, ICZ and MCZ to the OCZ of the host trees (Fig 2), indicating that diversity of vascular epiphytes shows a decreasing trend as we move up the trunk of host trees. This declining vertical gradient in epiphyte diversity may be related to the decrease in humidity and increase in ultraviolet radiation and photon flux density along the increasing canopy height of the hosts [13]. Tropical cloud forest in this study is located at the mountaintop, where the structure of crown layers is simple, with increased exposure to high ultraviolet radiation and wind. But the intensity of light decreases progressively from the canopy to the forest floor, whereas humidity progressively increases. Additionally, exposure to winds will naturally increase with tree height, further adding to the potential for epiphytes growing closer to the tops of trees to experience greater water evaporation. Thus, the lower light intensity and higher humidity in the understory compared to the crown layers [13] are more favorable for the growth of epiphytes. Our results are consistent with the research by Adriano & Mário [14], in which the abundance and species richness of epiphytes on the trunk were higher than those in the outer crown zone of host trees. Our results differ from those of Zhao et al. [10], who found that in a tropical montane forest, epiphyte diversity is the highest in the middle canopy. Collectively, this body of work suggestes that different critical environmental factors constrain epiphytic growth in these two types of forests: canopy soil in tropical montane forest, humidity and light in tropical cloud forest. Our study demonstrates that different tropical forest ecosystems have distinct patterns of vertical distribution of epiphytes, and underscores the unique ecology of tropical cloud forests.

Epiphyte abundance significantly differs, but epiphyte species richness does not differ among the five orientations when all the host trees collectively studied in tropical cloud forests (Fig 3), with the abundance and species richness of epiphytes encircling the trunk (all direction orientation) highest while both were lowest for north-oriented epiphytes. Our plots are located on the eastern slope, and wind often goes from the mountain ridges along the slopes. The lower epiphyte abundance and species richness of north-oriented epiphytes may be associated with the strong wind in the north which can cause epiphytes to experience higher water evaporation, as well as colder temperatures and lower irradiance. The higher epiphyte abundance and species richness of epiphytes encircling tree trunks (all direction orientation), however, suggests that microclimates around the host trees in tropical cloud forest generally favor the development of epiphyte communities. It is also possible that encircling epiphytes benefit from stronger growth on one side of the trunk that supports growth on less favourable sides. This may also explain why both epiphyte abundance and species richness do not significantly differ among the east, south, west and the north. We also find that both epiphyte abundance and species richness differ among the five orientations for each of six host trees, with inconsistent patterns for each orientation in each host tree (S7 Table). This is probably related to differences in morphological and physiological characteristics upon different orientations of host trees, including tree architecture [6], bark roughness [7], canopy soil chemistry [8], and branch inclination [9], as well as light penetration [13]. This result is also consistent with work by Tremblay and Castro [12], in which orchids were preferentially distributed on the northwestern side of the bole of hosts. The preference of epiphytes for a specific cardinal position on host trees shows that those wishing to promote the establishment of new epiphyte populations should consider this information to maximize epiphyte survivorship.

### Relationships between vascular epiphyte species richness and host tree diameter

As hypothesized, when all individuals with different diameters were taken together, epiphyte species richness and tree diameter are positively correlated across the six host tree species (Fig 4). What’s more, regression of breakpoints analysis based on the GLM supports a shift in the slope of this accumulation with increasing DBH. Across species, epiphyte species richness increases with increasing host diameter across all stages of host tree development in tropical cloud forests. Increases in epiphyte species richness with DBH may result directly from increased area for epiphyte colonization and growth coupled with increased exposure to epiphyte propagule rain over the course of a longer host tree life. This pattern may also result from resulting diversity in microenvironments available on larger tree trunks [13,18]. The fact that 200 of the host trees examined had fewer than five epiphytes suggests that host trees are not saturated and that potential exists for further epiphyte colonization. Therefore, our results are consistent with patterns observed by Laube and Zotz [41], that epiphyte diversity increased with increasing tree size. Our results reinforce the importance of large trees for the maintenance of vascular epiphytes; these large, mature trees play a critical role in maintaining the forest biodiversity in forest managements [42].

Interestingly, we found evidence that the slope of regressions of breakpoints examining the relationship between epiphyte species richness and tree diameter varied with tree diameter (Fig 4). The slope is high when the tree ontogeny is at an early stage but the accumulation of epiphyte species decelerates for as trees enter later ontogenetic stages (Fig 4). This suggests that there are two patterns for epiphyte colonization and growth on host trees in tropical cloud forests [18,23]. Several explanations are possible. First, it is possible that in the relatively early stages of tree growth, new habitat is exposed to epiphyte seed rain and, on average, colonization occurs more quickly, and decelerates as prime habitat on the host tree is captured. Second, and related to the first, the early stages of tree growth may be related to the generation of a larger number of relevant microsites that epiphytic species are adapted to, which encourages a wide spectrum of ephiphitic species, while later tree growth simply expands the area of these microsites, resulting in a slower accumulation of epiphytic species. Thirdly, there may be facilitative interactions among epiphyte species at early stages, as predicted within the stress-gradient hypothesis [31–33]; tree species within this habitat have already been shown to follow patterns expected under this theory [43]. The initial arrival of epiphytes may ameliorate the environments for the coming of new epiphyte species, contributing to an increased rate in epiphyte species richness at early stage of tree ontogeny. Similar to the second explanation, once tree growth provides all microsites in each zone, the accumulation of epiphyte species slows down, resulting in the slow accumulation of epiphytes on relatively old host trees. Our results are consistent to those of Flores-Palacios & García-Franco [22], who suggested that epiphyte species are saturated when the trees develop into adult stage, and that for mature trees, there should be no relationship between tree diameter and epiphyte species richness. Although saturation of epiphytes on host trees is expected at later stages of host tree ontogeny [44], we seldom find tree crowns that are completely covered by epiphytes during field work. The rate of colonization and growth for epiphytes is lower in the presence of low air temperature stress [44] (Fig 4); as such, it may be unreasonable to expect saturation in mature trees in tropical cloud forest. Future work should explore other potential influences on epiphyte colonization and survival, such as stresses related to temperature and wind. Our finding that ephiphyte richness increases with DBH for relatively small host trees contrasts with those of Taylor & Burns [23], who found no relationship between epiphyte species richness and host tree diameter for host trees below average diameter. However, this observation was consistent with our species specific explorations of this relationship (S8 Table). Regardless, our results do not support the suggestion that host trees at the early stage of development lack the architectural and physiological characteristics suitable for epiphyte establishment. Our results are also consistent with forest community succession theory [45], in that the early arrival of some epiphytes may facilitate the later establishment of new species.

## Conclusion

We demonstrate that tropical cloud forests are communities that host a diverse array of vascular epiphytes, and that these are dominated by orchids and pteridophytes. Both species richness and abundance of epiphytes significantly decreased from the lower to upper crown zones on host trees. Additionally, both epiphyte abundance and species richness did not differ among the eastern, southern, western and northern orientations for all collective host trees, but did differ among the these four orientations for individual host trees. When all six host species were considered together, vascular epiphyte species richness significantly increased with increasing host tree diameter, which is in contrast with studies predicting that epiphyte species richness had a neutral relationship with host tree size at later host stages [22]. We found that the rate at which epiphyte diversity increased with tree diameter was high at the early stages of host tree growth, but was lower at the later stages, contrasting with previous research that found no relationship between epiphyte richness and tree diameter at early stages [23].

## Supporting Information

S1 Table Vascular Epiphyte species composition and their distributions at crown zones and orientations upon host trees in tropical cloud forests in Hainan. (DOC)

S2 Table Effects of host tree height and host tree identities on epiphytic species richness/abundance, using the data of epiphytic species richness/abundance along different crown zones. (DOC)

S3 Table Differences in vascular epiphyte abundance and richness for each of the six host tree species along host tree height and among different host crown zone, using two-way ANOVAs. (DOC)

S4 Table Difference tests in vascular epiphyte abundance and richness for each of the six host tree species among the four host crown zones, using a one-way ANOVA. (DOC)

S5 Table Effects of host tree height and host tree identities on epiphytic species richness/abundance, using the data of epiphytic species richness/abundance along different orientations. (DOC)

S6 Table Differences in vascular epiphyte abundance and richness for each of the six host tree species along host tree height and among different host orientations, using two-way ANOVAs. (DOC)

S7 Table Difference tests in vascular epiphyte abundance and richness for each of the six host tree species among different epiphytic orientations, using a one-way ANOVA. (DOC)

S8 Table Parameters for relationships between vascular epiphyte species richness and DBH of host trees for each the six host tree species, using a generalized linear model. (DOC)
