# Supplementary material for: Vascular Epiphyte Diversity Differs with Host Crown Zone and Diameter, but Not Orientation in a Tropical Cloud Forest
Source: PLoS One. 2016 Jul 8;11(7):e0158548. doi: 10.1371/journal.pone.0158548 (PMC4938396; doi:10.1371/journal.pone.0158548)
Supplement: S1 Table — (DOC) [file pone.0158548.s001.doc]

**S1 Table.** Vascular epiphyte species composition and their distributions at crown zones and orientations upon host trees in tropical cloud forests in Hainan. TZ, ICZ, MZ and OCZ indicated trunk zone, inner crown zone, middle crown zone and outer crown zone of host trees, respectively. E, S, W, N and A indicated east, west, north and south directions upon host trees, respectively, as well as “all direction” where epiphytes grew around the host trees.

| Species | Family | Abundance | Host crown zone | Epiphytic orientation |
| --- | --- | --- | --- | --- |
| *Coelogyne fimbriata* | Orchidaceae | 305 | TZ,ICZ,MCZ,OCZ | E,S,W,N, A |
| *Bulbophyllum retusiusculum* | Orchidaceae | 204 | TZ,ICZ,MCZ,OCZ | E,S,W,N, A |
| *Pyrrosia eberhardtii* | Polypodiaceae | 149 | TZ,ICZ,MCZ,OCZ | E,S,W,N, A |
| *Pholidota chinensis* | Orchidaceae | 110 | TZ,ICZ,MCZ,OCZ | E,S,W,N, A |
| *Liparis delicatula* | Orchidaceae | 85 | TZ,ICZ,MCZ | E,S,W,N, A |
| *Eria obvia* | Orchidaceae | 82 | TZ,ICZ,MCZ,OCZ | E,S,W,N, A |
| *Dendrobium sinense* | Orchidaceae | 81 | TZ,ICZ,MCZ,OCZ | E,S,W,N,A |
| *Pholidota yunnanensis* | Orchidaceae | 78 | TZ,ICZ,MCZ,OCZ | E,S,W,N,A |
| *Humata repens* | Davalliaceae | 75 | TZ,ICZ,MCZ,OCZ | E,S,W,N, A |
| *Bulbophyllum ambrosia* | Orchidaceae | 55 | TZ,MCZ,ICZ,OCZ | E,S,W,N |
| *Lepidogrammitis rostrata* | Polypodiaceae | 47 | TZ,ICZ,MCZ,OCZ | E,S,W,N, A |
| *Schoenorchis gemmata* | Orchidaceae | 32 | TZ,ICZ,MCZ,OCZ | E,S,W,NA |
| *Eria thao* | Orchidaceae | 30 | TZ,ICZ,MCZ,OCZ | E,S,W,N,A |
| *Lepisorus thunbergianus* | Polypodiaceae | 22 | TZ,ICZ,MCZ,OCZ | E,S,W,N,A |
| *Epigeneium clemensiae* | Orchidaceae | 16 | TZ,ICZ,MCZ | E,S,W,N,A |
| *Dendrobium williamsonii* | Orchidaceae | 14 | TZ,ICZ,MCZ,OCZ | E,S,W |
| *Dendrobium densiflorum* | Orchidaceae | 13 | TZ,ICZ | E,S,W,N |
| *Bulbophyllum ledungense* | Orchidaceae | 9 | TZ,ICZ,MCZ | E,W,A |
| *Phymatopteris obtuse* | Polypodiaceae | 8 | TZ,ICZ | E,A,W |
| *Haplopteris flexuosa* | Vittariaceae | 6 | TZ,ICZ | A,S,W,E |
| *Cleisostoma paniculatum* | Orchidaceae | 6 | TZ,ICZ,OCZ | E,S,N |
| *Hoya griffithii* | Asclepiadaceae | 5 | TZ,ICZ,OCZ | N,E,S |
| *Eria pannea* | Orchidaceae | 3 | MCZ,ICZ | A,N |
| *Eria quinquelamellosa* | Orchidaceae | 3 | TZ,ICZ | E,S |
| *Ceratostylis hainanensis* | Orchidaceae | 3 | TZ,ICZ,MCZ | E,S |
| *Dendrobium nobile* | Orchidaceae | 2 | MCZ，ICZ | E,W |
| *Lycopodium japonicum* | Lycopodiaceae | 2 | TZ,ICZ | W,E |
| *Flickingeria angustifolia* | Orchidaceae | 1 | TZ | A |
| *Nephelaphyllum tenuiflorum* | Orchidaceae | 1 | TZ | W |
| *Arachnis labrosa* | Orchidaceae | 1 | MCZ | W |
| *Haplopteris amboinensis* | Vittariaceae | 1 | TZ | S |
| *Phymatopteris hainanensis* | Polypodiaceae | 1 | TZ | W |
| *Neottopteris nidus* | Aspleniaceae | 1 | TZ | N |
| *Pseudodrynaria coronans* | Drynariaceae | 1 | MCZ | A |
| *Selaginella tamariscina* | Selaginellaceae | 1 | TZ | W |
