# Supplementary material for: Vascular Epiphyte Diversity Differs with Host Crown Zone and Diameter, but Not Orientation in a Tropical Cloud Forest
Source: PLoS One. 2016 Jul 8;11(7):e0158548. doi: 10.1371/journal.pone.0158548 (PMC4938396; doi:10.1371/journal.pone.0158548)
Supplement: S2 Table — (DOC) [file pone.0158548.s002.doc]

**S2 Table.** Effects of host tree height and host tree identities on epiphytic species richness/abundance, using the data of epiphytic species richness/abundance along different crown zones. Host tree height was taken as a fixed effect, while host tree species identity was considered as random effects in the linear mixed-effects model. Models were fit by maximum likelihood, and *student* t-tests were used to assess the significance of the fixed effect in the model. Est. indicated the parameter estimate. Std. Err. indicated standard errors. Var. indicated variance of species richness and abundance of vascular epiphytes explained by host identity and residuals.

| Vascular epiphyte abundance | | | | | |  | Vascular epiphyte species richness | | | | |
| --- | --- | --- | --- | --- | --- | --- | --- | --- | --- | --- | --- |
|  | Est. | Std. Err. | *t* | *P* | Var. (%) |  | Est. | Std. Err. | *t* | *P* | Var. (%) |
| Intercept | 1.01 | 0.18 | 5.73 | <0.001 | — |  | 1.04 | 0.17 | 6.20 | < 0.001 | — |
| Host identity | — | 0.09 | — | — | 5.86 |  | — | 0.12 | — | — | 7.85 |
| Host height | 0.006 | 0.02 | 0.29 | 0.76 | — |  | -0.006 | 0.02 | -0.32 | 0.74 | — |
| Residuals | — | 1.46 | — | — | 94.14 |  | — | 1.36 | — | — | 92.19 |
