# Supplementary material for: Vascular Epiphyte Diversity Differs with Host Crown Zone and Diameter, but Not Orientation in a Tropical Cloud Forest
Source: PLoS One. 2016 Jul 8;11(7):e0158548. doi: 10.1371/journal.pone.0158548 (PMC4938396; doi:10.1371/journal.pone.0158548)
Supplement: S3 Table — (DOC) [file pone.0158548.s003.doc]

**S3 Table.** Differences in vascular epiphyte abundance and richness for each of the six host tree species along host tree height and among different host crown zone, using two-way ANOVAs. Cyc_dis, Ter_gym, Ill_ter, Eng_rox, Syz_bux and Dis_rac indicated *Cyclobalanopsis disciformis*, *Ternstroemia gymnanthera*, *Illicium ternstroemioides*, *Engelhardtia roxburghiana*, *Syzygium buxifolium*, *Distylium racemosum*, respectively.

|  | Vascular epiphyte abundance | | | | |  | Vascular epiphyte species richness | | | |
| --- | --- | --- | --- | --- | --- | --- | --- | --- | --- | --- |
|  |  | df | Sum Square | *F* | *P* |  | df | Sum Square | *F* | *P* |
| Cyc_dis | Host height | 1 | 0.28 | 0.23 | 0.63 |  | 1 | 0.02 | 0.03 | 0.87 |
|  | Crown zone | 3 | 14.93 | 4.03 | 0.01 |  | 3 | 13.86 | 5.38 | 0.003 |
|  | Host height: Crown zone | 3 | 4.89 | 1.32 | 0.28 |  | 3 | 1.73 | 0.67 | 0.57 |
|  | Residuals | 48 | 59.25 |  |  |  | 48 | 41.25 |  |  |
| Ter_gym | Host height | 1 | 0.17 | 0.12 | 0.73 |  | 1 | 0 | 0.001 | 0.97 |
|  | Crown zone | 3 | 48.05 | 11.99 | <0.001 |  | 3 | 39.00 | 9.06 | <0.001 |
|  | Host height: Crown zone | 3 | 0.50 | 0.13 | 0.95 |  | 3 | 0.69 | 0.16 | 0.93 |
|  | Residuals | 52 | 69.47 |  |  |  | 52 | 74.64 |  |  |
| Ill_ter | Host height | 1 | 0.89 | 0.74 | 0.39 |  | 1 | 0.07 | 0.10 | 0.76 |
|  | Crown zone | 3 | 34.52 | 9.60 | <0.001 |  | 3 | 23.08 | 11.21 | <0.001 |
|  | Host height: Crown zone | 3 | 2.84 | 0.95 | 0.51 |  | 3 | 0.34 | 0.17 | 0.92 |
|  | Residuals | 44 | 52.74 |  |  |  | 44 | 30.21 |  |  |
| Eng_rox | Host height | 1 | 0.09 | 0.08 | 0.78 |  | 1 | 0.03 | 0.04 | 0.85 |
|  | Crown zone | 3 | 43.41 | 13.55 | <0.001 |  | 3 | 39.57 | 14.01 | <0.001 |
|  | Host height: Crown zone | 3 | 0.66 | 0.21 | 0.89 |  | 3 | 0.42 | 0.15 | 0.93 |
|  | Residuals | 60 | 64.08 |  |  |  | 60 | 56.49 |  |  |
| Syz_bux | Host height | 1 | 0.01 | 0.01 | 0.92 |  | 1 | 0.61 | 0.81 | 0.37 |
|  | Crown zone | 3 | 9.88 | 3.64 | 0.02 |  | 3 | 8.03 | 3.54 | 0.02 |
|  | Host height: Crown zone | 3 | 0.54 | 0.20 | 0.90 |  | 3 | 0.09 | 0.04 | 0.99 |
|  | Residuals | 104 | 94.06 |  |  |  | 104 | 78.55 |  |  |
| Dis_rac | Host height | 1 | 0.40 | 0.25 | 0.62 |  | 1 | 0.60 | 0.42 | 0.52 |
|  | Crown zone | 3 | 486.70 | 109.74 | <0.001 |  | 3 | 419.50 | 99.93 | <0.001 |
|  | Host height: Crown zone | 3 | 6.10 | 1.38 | 0.25 |  | 3 | 4.60 | 1.09 | 0.35 |
|  | Residuals | 388 | 573.60 |  |  |  | 388 | 542.90 |  |  |
