# Supplementary material for: Vascular Epiphyte Diversity Differs with Host Crown Zone and Diameter, but Not Orientation in a Tropical Cloud Forest
Source: PLoS One. 2016 Jul 8;11(7):e0158548. doi: 10.1371/journal.pone.0158548 (PMC4938396; doi:10.1371/journal.pone.0158548)
Supplement: S4 Table — (DOC) [file pone.0158548.s004.doc]

**S4 Table.** Difference tests in vascular epiphyte abundance and richness for each of the six host tree species among the four host crown zones, using a one-way ANOVA.

| Vascular epiphyte abundance | | | Vascular epiphyte species richness | |
| --- | --- | --- | --- | --- |
| *Distylium racemosum* | *F_(3, 388)_ =* 110.00 | *P <* 0.001 | *F_(3, 388)_ =* 100.20 | *P* < 0.001 |
| *Syzygium buxifolium* | *F*_(3, 108)_ = 3.76 | *P* = 0.013 | *F*_(3, 108)_ = 3.65 | *P* = 0.015 |
| *Engelhardtia roxburghiana* | *F*_(3, 64)_ = 14.29 | *P <* 0.001 | *F*_(3, 64)_ = 14.83 | *P* < 0.001 |
| *Ternstroemia gymnanthera* | *F*_(3, 56)_ = 12.79 | *P* < 0.001 | *F*_(3, 56)_ = 19.02 | *P* < 0.001 |
| *Cyclobalanopsis disciformis* | *F*_(3, 52)_ = 4.02 | *P* = 0.01 | *F*_(3, 52)_ = 5.59 | *P* = 0.002 |
| *Illicium ternstroemioides* | *F*_(3, 48)_ = 14.47 | *P* < 0.001 | *F*_(3, 48)_ = 12.06 | *P* < 0.001 |
