# Supplementary material for: Vascular Epiphyte Diversity Differs with Host Crown Zone and Diameter, but Not Orientation in a Tropical Cloud Forest
Source: PLoS One. 2016 Jul 8;11(7):e0158548. doi: 10.1371/journal.pone.0158548 (PMC4938396; doi:10.1371/journal.pone.0158548)
Supplement: S6 Table — (DOC) [file pone.0158548.s006.doc]

**S6 Table.** Differences in vascular epiphyte abundance and richness for each of the six host tree species along host tree height and among different host orientations, using two-way ANOVAs. Cyc_dis, Ter_gym, Ill_ter, Eng_rox, Syz_bux and Dis_rac indicated *Cyclobalanopsis disciformis*, *Ternstroemia gymnanthera*, *Illicium ternstroemioides*, *Engelhardtia roxburghiana*, *Syzygium buxifolium*, *Distylium racemosum*, respectively.

|  | Vascular epiphyte abundance | | | | |  | Vascular epiphyte species richness | | | |
| --- | --- | --- | --- | --- | --- | --- | --- | --- | --- | --- |
|  |  | df | Sum Square | *F* | *P* |  | df | Sum Square | *F* | *P* |
| Cyc_dis | Host height | 1 | 0.47 | 0.44 | 0.51 |  | 1 | 0.06 | 0.10 | 0.76 |
|  | Orientation | 4 | 39.23 | 9.20 | <0.001 |  | 4 | 19.94 | 8.62 | <0.001 |
|  | Host height: Orientation | 4 | 3.20 | 0.75 | 0.56 |  | 4 | 3.90 | 1.69 | 0.17 |
|  | Residuals | 60 | 63.98 |  |  |  | 60 | 34.69 |  |  |
| Ter_gym | Host height | 1 | 0.24 | 0.10 | 0.75 |  | 1 | 0.52 | 0.41 | 0.53 |
|  | Orientation | 4 | 29.65 | 3.20 | 0.02 |  | 4 | 14.53 | 2.82 | 0.03 |
|  | Host height: Orientation | 4 | 4.59 | 0.50 | 0.74 |  | 4 | 3.12 | 0.61 | 0.66 |
|  | Residuals | 65 | 150.51 |  |  |  | 65 | 83.82 |  |  |
| Ill_ter | Host height | 1 | 0.71 | 0.64 | 0.43 |  | 1 | 0.58 | 0.68 | 0.41 |
|  | Orientation | 4 | 27.94 | 6.24 | <0.001 |  | 4 | 22.68 | 6.59 | <0.001 |
|  | Host height: Orientation | 4 | 1.58 | 0.35 | 0.84 |  | 4 | 1.47 | 0.43 | 0.79 |
|  | Residuals | 55 | 61.55 |  |  |  | 55 | 47.33 |  |  |
| Eng_rox | Host height | 1 | 0.07 | 0.04 | 0.85 |  | 1 | 0.12 | 0.12 | 0.73 |
|  | Orientation | 4 | 47.69 | 6.20 | <0.001 |  | 4 | 20.42 | 5.04 | 0.001 |
|  | Host height: Orientation | 4 | 1.02 | 0.13 | 0.97 |  | 4 | 1.01 | 0.25 | 0.91 |
|  | Residuals | 75 | 144.20 |  |  |  | 75 | 76.04 |  |  |
| Syz_bux | Host height | 1 | 0.01 | 0.007 | 0.93 |  | 1 | 1.32 | 2.20 | 0.14 |
|  | Orientation | 4 | 15.71 | 3.60 | 0.008 |  | 4 | 11.47 | 4.78 | 0.001 |
|  | Host height: Orientation | 4 | 9.80 | 2.25 | 0.07 |  | 4 | 2.13 | 0.89 | 0.47 |
|  | Residuals | 130 | 141.87 |  |  |  | 130 | 78.05 |  |  |
| Dis_rac | Host height | 1 | 0.30 | 0.24 | 0.63 |  | 1 | 0.10 | 0.10 | 0.75 |
|  | Orientation | 4 | 102.00 | 18.09 | <0.001 |  | 4 | 66.30 | 16.02 | <0.001 |
|  | Host height: Orientation | 4 | 37.30 | 6.61 | <0.001 |  | 4 | 19.50 | 4.70 | 0.001 |
|  | Residuals | 485 | 683.7 |  |  |  | 485 | 501.80 |  |  |
