# Supplementary material for: Vascular Epiphyte Diversity Differs with Host Crown Zone and Diameter, but Not Orientation in a Tropical Cloud Forest
Source: PLoS One. 2016 Jul 8;11(7):e0158548. doi: 10.1371/journal.pone.0158548 (PMC4938396; doi:10.1371/journal.pone.0158548)
Supplement: S7 Table — (DOC) [file pone.0158548.s007.doc]

**S7 Table. Difference tests in vascular epiphyte abundance and richness for each of the six host tree species among different epiphytic orientations, using a one-way ANOVA.**

| Vascular epiphyte abundance | | | Vascular epiphyte species richness | |
| --- | --- | --- | --- | --- |
| *Distylium racemosum* | *F*_(4, 490)_ = 17.73 | *P <* 0.001 | *F*_(4, 490)_ = 15.58 | *P* < 0.001 |
| *Syzygium buxifolium* | *F*_(4, 135)_ = 5.12 | *P* < 0.001 | *F*_(4, 135)_ = 4.75 | *P* =0.001 |
| *Engelhardtia roxburghiana* | *F*_(4, 80)_ = 4.97 | *P* < 0.001 | *F*_(4, 80)_ = 5.29 | *P* < 0.001 |
| *Ternstroemia gymnanthera* | *F*_(4, 70)_ = 2.48 | *P* = 0.052 | *F*_(4, 70)_ = 2.91 | *P* = 0.028 |
| *Cyclobalanopsis disciformis* | *F*_(4, 65)_ = 8.30 | *P* < 0.001 | *F*_(4, 65)_ = 8.39 | *P* < 0.001 |
| *Illicium ternstroemioides* | *F*_(4, 60)_ = 8.01 | *P* < 0.001 | *F*_(4, 60)_ = 6.89 | *P* < 0.001 |
