# Supplementary material for: Vascular Epiphyte Diversity Differs with Host Crown Zone and Diameter, but Not Orientation in a Tropical Cloud Forest
Source: PLoS One. 2016 Jul 8;11(7):e0158548. doi: 10.1371/journal.pone.0158548 (PMC4938396; doi:10.1371/journal.pone.0158548)
Supplement: S8 Table — (DOC) [file pone.0158548.s008.doc]

**S8 Table.** Parameters for relationships between vascular epiphyte species richness and DBH of host trees for each the six host tree species, using a generalized linear model.

| Host tree species | df | DBH | Null deviance | AIC |
| --- | --- | --- | --- | --- |
| *Distylium racemosum* | 99 | 0.14 | 99.90 | 403.36 |
| *Syzygium buxifolium* | 27 | 0.18 | 29.09 | 97.69 |
| *Cyclobalanopsis disciformis* | 13 | 0.80 | 5.91 | 48.99 |
| *Illicium ternstroemioides* | 12 | 0.57 | 7.00 | 49.24 |
| *Ternstroemia gymnanthera* | 14 | 0.12 | 13.47 | 61.39 |
| *Engelhardtia roxburghiana* | 16 | 0.09 | 7.46 | 60.44 |
